# Icariin Promotes the Migration of BMSCs In Vitro and In Vivo via the MAPK Signaling Pathway

**DOI:** 10.1155/2018/2562105

**Published:** 2018-09-18

**Authors:** Feng Jiao, Wang Tang, He Huang, Zhaofei Zhang, Donghua Liu, Hongyi Zhang, Hui Ren

**Affiliations:** ^1^Guangzhou Hospital of Integrated Traditional and Western Medicine, China; ^2^Guangzhou University of Chinese Medicine, China; ^3^The First Affiliated Hospital of Guangzhou University of Traditional Chinese Medicine, China

## Abstract

Bone marrow-derived mesenchymal stem cells (BMSCs) are widely used in tissue engineering for regenerative medicine due to their multipotent differentiation potential. However, their poor migration ability limits repair effects. Icariin (ICA), a major component of the Chinese medical herb Herba Epimedii, has been reported to accelerate the proliferation, osteogenic, and chondrogenic differentiation of BMSCs. However, it remains unknown whether ICA can enhance BMSC migration, and the possible underlying mechanisms need to be elucidated. In this study, we found that ICA significantly increased the migration capacity of BMSCs, with an optimal concentration of 1 *μ*mol/L. Moreover, we found that ICA stimulated actin stress fiber formation in BMSCs. Our work revealed that activation of the MAPK signaling pathway was required for ICA-induced migration and actin stress fiber formation. In vivo, ICA promoted the recruitment of BMSCs to the cartilage defect region. Taken together, these results show that ICA promotes BMSC migration in vivo and in vitro by inducing actin stress fiber formation via the MAPK signaling pathway. Thus, combined administration of ICA with BMSCs has great potential in cartilage defect therapy.

## 1. Introduction

Osteoarthritis (OA), known as degenerative arthritis or joint disease, may lead to the loss of cartilage [1, 2]. Lack of vessels, nerves, and local progenitor cells leads to difficulty in repairing cartilage. With the development of cell therapies, cell-based repair, which includes treatments with chondrocytes and bone marrow-derived mesenchymal stem cells (BMSCs), has recently attracted considerable attention from researchers. Although autologous chondrocyte implantation for the cartilage treatment is a practical solution, the limited sources and dedifferentiation of chondrocytes cultured in vitro restrict its application [3, 4]. In contrast, BMSCs are easy to obtain, have abundant sources, and exhibit strong reproductive activity. BMSCs can be directed to differentiate into many types of cells damaged by disease under certain conditions. Moreover, BMSCs can secrete active components that promote wound healing [5, 6]. However, BMSCs must successfully migrate to the wound to participate in repair processes. The low recruitment of BMSCs to target tissue deters their repair effect [7, 8]. Based on all these characteristics, enhancing the migration ability of BMSCs may be a promising research direction for treating cartilage defects.

Cell-based repair involves migration of stem cells from the sites where they colonize to the wound. The process of migration is regulated by several distinct but interacting signaling pathways. Among these, the mitogen-activated protein kinase (MAPK) signaling pathway has been widely researched [9, 10] and has been confirmed to regulate microtubules and actin filaments; the latter of which can produce pushing (protrusive) forces or pulling (contractile) forces that are particularly important for whole-cell migration [11–13]. These findings provide new directions for the medicine screening.

Herba Epimedii (HEP) is a widely used traditional Chinese herb in the treatment of OA [14]. Icariin (ICA), the major pharmacologically active component of HEP, was proven to be an efficient accelerator of cartilage tissue engineering. ICA can accelerate the formation of cartilage matrix and chondroid tissue [15, 16]. Moreover, it has been found that ICA exerts multiple effects on BMSCs by activating MAPK signal pathway, including its ability to promote the proliferation and osteogenic, chondrogenic, and adipogenic differentiation [17–19]. Nonetheless, whether ICA has the potential to promote the migration of BMSCs and whether the possible underlying mechanism occurs via the MAPK signaling pathway remain unclear.

In this study, we assessed the effect of ICA on BMSC migration and its underlying mechanisms. In addition, BMSCs were injected via the ear vein of rabbits with knee articular cartilage defects to investigate the effect of ICA on BMSC migration in vivo.

## 2. Materials and Methods

### 2.1. Materials

Three-month-old female New Zealand rabbits (2 ± 0.5 kg) were purchased from the Center of Experimental Animals at Guangzhou University of Chinese Medicine. All animals were treated according to the animal guidelines of Guangzhou University of Chinese Medicine. Experimental measurements were carried out in the Laboratory of Orthopaedics and Traumatology of Chinese Medicine of the Lingnan Medical Research Center at Guangzhou University of Chinese Medicine.

### 2.2. Cell Culture

BMSCs were isolated and cultured from the bone marrow of 3-month-old female New Zealand rabbits. The cells were cultured in alpha minimum essential medium (MEM) containing 10% fetal bovine serum and 1% penicillin-streptomycin in an incubator at 5% CO_2_ at 37°C. The culture flask was washed with phosphate-buffered saline (PBS) to remove nonadherent cells after 72 h. When grown to 80–90% confluence, the BMSCs were trypsinized and passaged, and the medium was replaced every 2 days.

To determine whether the isolated BMSCs possess multipotent differentiation ability in vitro, BMSCs at passage 3 were grown in osteogenic medium consisting of alpha MEM containing 10% fetal bovine serum, 1% penicillin-streptomycin, 0.2% ascorbate, 1% *β*-glycerophosphate, and 0.01% dexamethasone or chondrogenic medium containing 0.01% dexamethasone, 0.3% ascorbate, 1% insulin-transferrin-selenium (ITS) + supplement, 0.1% sodium pyruvate, 0.1% proline, and 1% TGF-*β*3 for 14 days. The medium was replaced every three days. The cells were then washed with PBS and then fixed with 4% paraformaldehyde for 10 min. Osteogenic cells were stained with alkaline phosphatase and alizarin red. Chondrogenic cells were stained with alcian blue. Photographs were taken using an inverted microscope with a camera.

### 2.3. Cell Counting Kit-8 (CCK-8) Assay

Cell proliferation was measured using the CCK-8 assay. BMSCs at passage 4 were seeded in 96-well plates at a density of 5 × 10^3^ cells with four replicates for each group. The groups were as follows: 0 *μ*M ICA, 0.01 *μ*M ICA, 0.1 *μ*M ICA, 1 *μ*M ICA, 10 *μ*M ICA, and 100 *μ*M ICA. Cell proliferation was measured at 12, 24, 36, 48, 72, and 76 h after incubation. The CCK-8 solution was changed after the different intervals of incubation. Then, the plates were then incubated at 37°C for an additional 40 min. The optical density was determined at 520 nm using a microplate reader.

### 2.4. Scratch Wound-Healing Assay

BMSCs at passage 4 were seeded in 6-well plates at a density of 2 × 10^5^ cells per well. When the cells grew to 95% confluence, the medium was aspirated out of the well, and cells were serum-starved for 12 h. A scratch wound was created with a micropipette tip, and the cells were washed twice with PBS to remove cellular debris and floating cells, followed by incubation with vehicle. The control group was treated with culture medium, and the experimental groups were treated with different doses of ICA. The remaining wound area was observed and photographed using an inverted microscope with a camera at 0, 12, and 24 h.

### 2.5. Transwell Migration Assay

BMSCs were subjected to serum deprivation for 12 h before being cultured on a polycarbonate porous membrane insert with 8 *μ*m pores (Corning Costar, Shanghai, China). The cell density was adjusted to 1 × 10^6^ cells/mL with alpha MEM containing fetal bovine serum. The cells were divided into the following groups: (1) control group (0 *μ*M ICA in both the upper and lower chambers); (2) 0.1 *μ*M ICA group (upper chamber: 0 *μ*M ICA, lower chamber: 0.1 *μ*M ICA); (3) 1 *μ*M ICA group (upper chamber: 0 *μ*M ICA, lower chamber: 1 *μ*M ICA); and (4) 10 *μ*M group (upper chamber: 0 *μ*M ICA, lower chamber: 10 *μ*M ICA). One hundred microliters of cell suspension were added to the upper chamber, and alpha MEM was added to the lower chamber. The cells were allowed to migrate for 24 h; after which, the polycarbonate membrane was removed, fixed with 70% ethanol for 30 min, and stained with 0.1% crystal violet for 30 min. BMSCs were counted under a fluorescence microscope.

### 2.6. Rhodamine-Phalloidin Staining

BMSCs were grown in 24-well plates with glass coverslips at a density of 100 cells per well. BMSCs in the control group were treated with alpha MEM, while those in the experimental group were incubated with ICA. After treatment, the cells were washed with PBS, fixed with 4% paraformaldehyde for 10 min, washed with PBS, permeabilized with 0.5% Triton X-100 for 5 min, and incubated with 100 nM rhodamine-phalloidin prepared in 1% bovine serum albumin (BSA). Cells were counterstained with Prolong Gold AntiFade Reagent with DAPI for 30 seconds after washing with PBS. Photographs were taken with a confocal laser scanning microscope.

### 2.7. Western Blotting

After incubation in growth medium or growth medium containing 1 *μ*M ICA for 30, 60, and 120 min, cells were washed three times with cold PBS and suspended in 60 *μ*L of cell lysis buffer containing protease inhibitors (Beyotime, China). The suspension was centrifuged at 15,000 rpm for 20 min at 4°C, and the supernatant was reserved. The concentration of protein in the sample was measured by a BCA protein quantitation kit, and 10% sodium dodecyl sulfate–polyacrylamide gel electrophoresis (SDS-PAGE) was used to separate aliquots of lysates containing an equal amount of protein (20 *μ*g) followed by transfer onto polyvinylidene fluoride (PVDF) membranes. The membranes were washed with Tris-buffered saline (TBST), blocked for 1 h at room temperature in TBST containing 5% dry milk, and incubated overnight with specific primary antibodies (phospho-p38, phospho-ERK1/2, and phospho-JNK) at 4°C. Next, the membranes were incubated with secondary antibodies for 1 h at room temperature. Blots were visualized using a standard enhanced chemiluminescence system.

### 2.8. Immunofluorescence Assay

BMSCs at passage 3 were seeded in 12-well culture plates. When the cells grew to 60% confluence, the medium was aspirated from the plates and replaced with 10 *μ*M 5′-bromo-2-deoxyuridine (BrdU) for 48 h. After the media were aspirated, the cells were covered completely with cold 70% ethanol and fixed for 5 min at room temperature. The cells were then blocked with 5% normal goat serum and incubated with BrdU antibody overnight at 4°C. After rinsing three times with PBS for 5 min each, the cells were incubated in fluorochrome-conjugated secondary antibody diluted in Antibody Dilution Buffer for 1 h at room temperature in the dark. In addition, sufficient Prolong Gold AntiFade Reagent with DAPI was applied to cover cells in the 12-well culture plates. Fluorescence microscopy was employed to observe the rate of BrdU labeling.

### 2.9. Cartilage Defect Model

Fifteen three-month-old healthy female New Zealand white rabbits (2.5 ± 0.3 kg) were randomly divided into the normal control group, BMSC group, and ICA + BMSC group. After anesthesia, the knee joint was exposed, and a full-thickness cylindrical cartilage defect of 4 mm in diameter and 3 mm in depth (reaching the bone marrow exude) was created in the patellar groove using a standard-size stainless steel biopsy punch. In addition, 4 × 10^5^ U penicillin was intramuscularly injected after the operation. Two hours after surgery, rabbits in the experimental group were injected with BMSCs that had been incubated with ICA for 72 h, and rabbits in the BMSC group were injected with BMSCs without ICA pretreatment via the ear vein; the normal control group was injected with PBS. The BMSCs were all labeled with BrdU before injection. The rabbits were housed in separate hutches and allowed to move freely.

### 2.10. Immunohistochemical Staining of BrdU

The regenerated tissues of all groups were collected 4 weeks after surgery. The sample was fixed in 4% paraformaldehyde for 48 h, decalcified in 10% EDTA for 4 weeks until a needle could impale the tissues, paraffin embedded, and sectioned. After the sections were dewaxed and rehydrated, the slides were submersed in 1× citrate unmasking solution and heated in a microwave until boiling, after which they were maintained for 10 min at a subboiling temperature (95–98°C). After cooling on a bench top for 30 min and three PBS washes for 5 min each, the sections were incubated in 3% hydrogen peroxide for 10 min. The solution was then replaced with BrdU primary antibody for overnight incubation at 4°C. The sections were washed again and incubated with secondary antibody. Then, the sections were washed with PBS and coverslipped. Photographs were taken using an inverted phase contrast microscope. Positively stained BMSCs were quantified in three random areas of each section.

### 2.11. Statistical Analysis

Statistical analyses were performed using the SPSS 24.0 software. The results were presented as the mean ± standard deviation. Differences among groups were tested by one-way analysis of variance (ANOVA). *P* < 0.05 was considered to indicate a statistically significant difference.

## 3. Results

### 3.1. Identification of BMSCs

**Figure 1:**
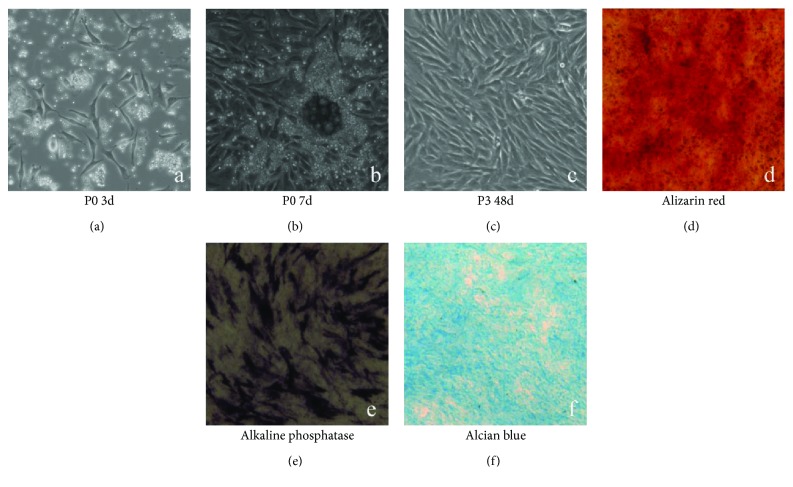
Characteristics of rabbit BMSCs. (a) Few BMSCs grew via static adherence cultured in growth medium in the primary phase. (b) The growth of BMSCs from embryoid body (EB) formation, reaching 80% confluence at day 7. (c) BMSCs at passage 3, reaching confluence 90% after incubation for 48 h. (d) Osteoplastic differentiation revealed by alizarin red staining after 2 weeks. (e) Osteoplastic differentiation revealed by alkaline phosphatase staining after 2 weeks. (f) Chondrogenic differentiation revealed by alcian blue staining after 2 weeks.

BMSCs were obtained from rabbits, and their multipotent differentiation ability was detected by osteoplastic and chondrogenic differentiation. BMSCs developed into osteoblasts and chondrocytes after incubation with differentiation solution for 14 days. Osteoplastic cells were detected by alizarin red and alkaline phosphatase, and chondrogenic cells were detected by alcian blue staining. These results showed that many cells remained BMSCs after three generations of subculture (Figure 1).

### 3.2. Effect of ICA on the Proliferation of BMSCs

**Figure 2:**
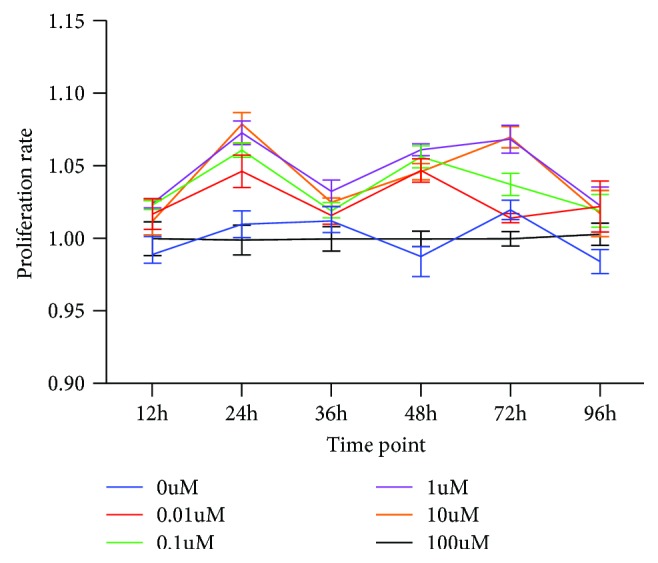
Effect of ICA on the proliferation of BMSCs. BMSCs were treated with various concentrations of ICA (0, 0.01, 0.1, 1, 10, and 100 *μ*M) for 12, 24, 36, 48, 72, and 96 h. The proliferation rate of BMSCs was assessed by the CCK-8 assay.

To investigate the effect of ICA on BMSC proliferation, we added different doses of ICA and then measured cell proliferation by the CCK-8 assay after 12, 24, 36, 48, 72, and 96 h. As shown in Figure 2, ICA did not noticeably promote BMSC proliferation. There were no statistically significant differences among the groups (Figure 2).

### 3.3. ICA Accelerates the Migration of BMSCs

**Figure 3:**
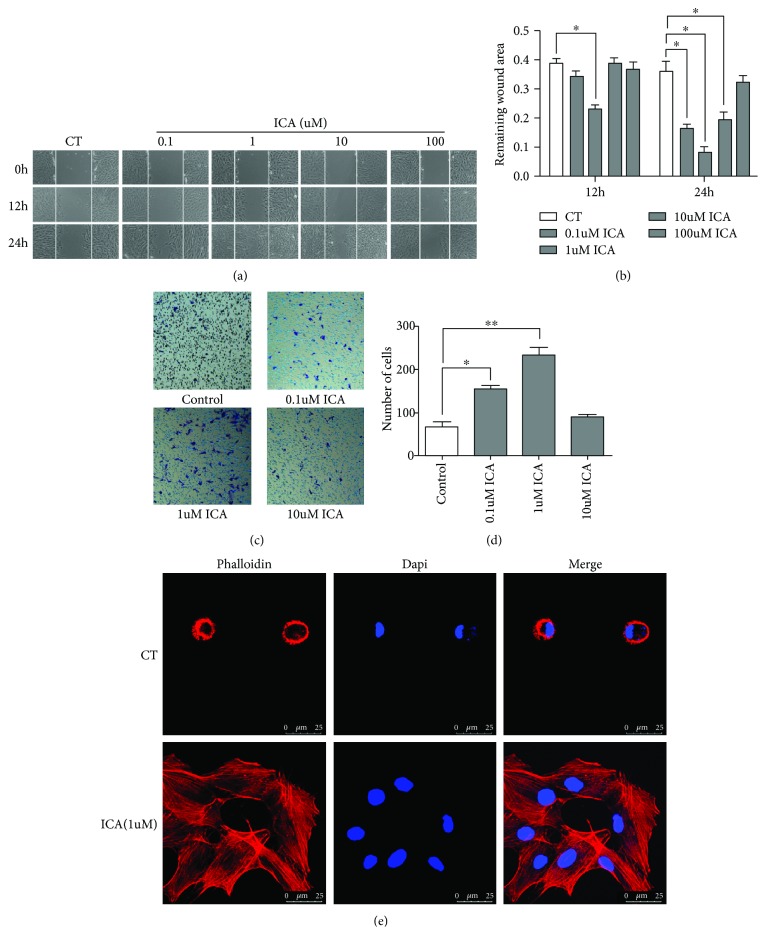
ICA promotes the migration of BMSCs in vitro. (a) Scratch wound-healing assay of BMSCs treated with ICA (0, 0.1, 1, 10, and 100 *μ*M). Phase contrast images were captured after 12 and 24 h. (b) Quantitative analysis of the remaining wound area in Figure 3(a). Three random fields of each group were selected, and the remaining area of wound was measured using Image J. (c) Transwell migration assay of BMSCs treated with ICA (0.1, 1, and 10 *μ*M). The number of BMSCs in the outside bottom chamber was calculated. (d) Quantitative analysis of the migrated cells. The results are shown as the mean value of 5 random fields. (e) ICA stimulated actin stress fiber formation of BMSCs. Data are shown as the mean ± SD of three independent experiments. ^∗^*P* < 0.05, ^∗∗^*P* < 0.01 compared with the group control.

The ability of ICA to promote BMSC migration was examined through the wound-healing assay and Transwell migration assay. In the wound-healing assay, many BMSCs in the groups treated with 0.1, 1, and 10 *μ*M ICA but few cells in the control group migrated to the scratch wound at 12 and 24 h after creating the scratch. Among all these groups, the remaining wound area in the group treated with 1 *μ*M ICA was the smallest, and the difference was statistically significant (*P* < 0.05). However, BMSC migration in the 100 *μ*M ICA group was not noticeably promoted compared with that in the control group (Figures 3(a) and 3(b)). As shown in Figure 3(c), the number of cells that migrated to the lower chamber in the 0.1 and 1 *μ*M ICA groups was much higher than that in the control group (*P* < 0.05). Although there was no significant difference in BMSC migration between the 1 *μ*M and 0.1 *μ*M ICA groups, more migrating cells were observed after culture with 1 *μ*M ICA than with 0.1 *μ*M ICA. These results show that 1 *μ*M ICA stimulates BMSC migration.

### 3.4. ICA Promotes the Migration of BMSCs Probably by Stimulating Actin Stress Fiber Formation

The cytoskeleton is a network of fibers composed of proteins contained within the cytoplasm in all cells of all domains of life, most notably in eukaryotic cells. The cytoskeleton of eukaryotes has three major components: microfilaments, microtubules, and intermediate filaments. By contrast, intermediate filaments consist of actin protein, which is the primary force-generating machinery in the cell and can produce pushing forces that can power diverse motility processes. To study the effect of ICA on actin proteins in BMSCs, rhodamine-phalloidin was used to stain actin protein. After ICA treatment (1 *μ*M), the formation of intermediate filaments was apparently increased compared with that in the control group (Figure 3(e)).

### 3.5. ICA Upregulates Protein Expression of the MAPK Signaling Pathway

**Figure 4:**
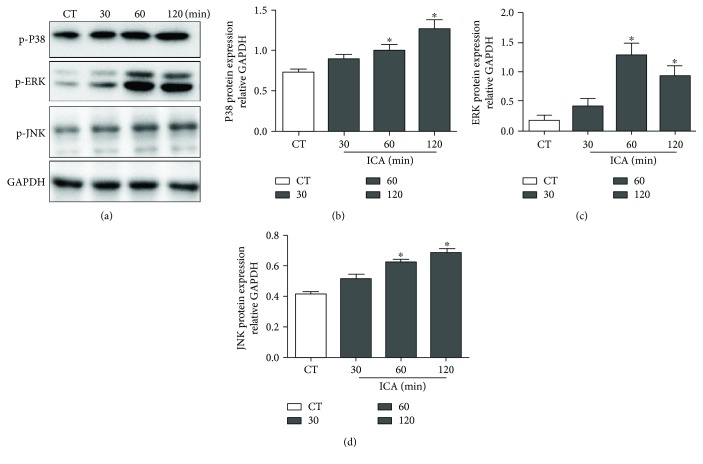
ICA upregulates the protein expression of the MAPK signaling pathway. BMSCs were treated with ICA for 30, 60, and 120 min. Total protein extracts were prepared, and the phosphorylation levels of P38, ERK1/2, and JNK were detected by Western blot. The experiment was repeated at least three times to verify the result. (a) Representative bands of P38, ERK 1/2, and P38. The internal reference was GAPDH. (b, c, d) Densitometric analysis of immunoblotting of phosphorylated-P38, ERK1/2, and JNK compared by one-way ANOVA. Data are shown as the mean ± SD of three independent experiments. ^∗^*P* < 0.05 compared with the group control.

There are many signaling pathways involved in BMSC migration, but the MAPK signaling pathway is the most crucial. To examine whether ICA can upregulate the MAPK signaling pathway, cells were treated with ICA (1 *μ*M) for 30, 60, or 120 min, and the expressions of p-P38, extracellular regulated kinase (ERK or p42/p44 MAPK), and jun amino-terminal kinases/stress-activated protein kinase (JNK) were detected by Western blot. We found that p-P38, ERK, and JNK were increased after ICA treatment (Figure 4).

### 3.6. MAPK Signaling Pathway Participates in the Migration of BMSCs Induced by ICA

**Figure 5:**
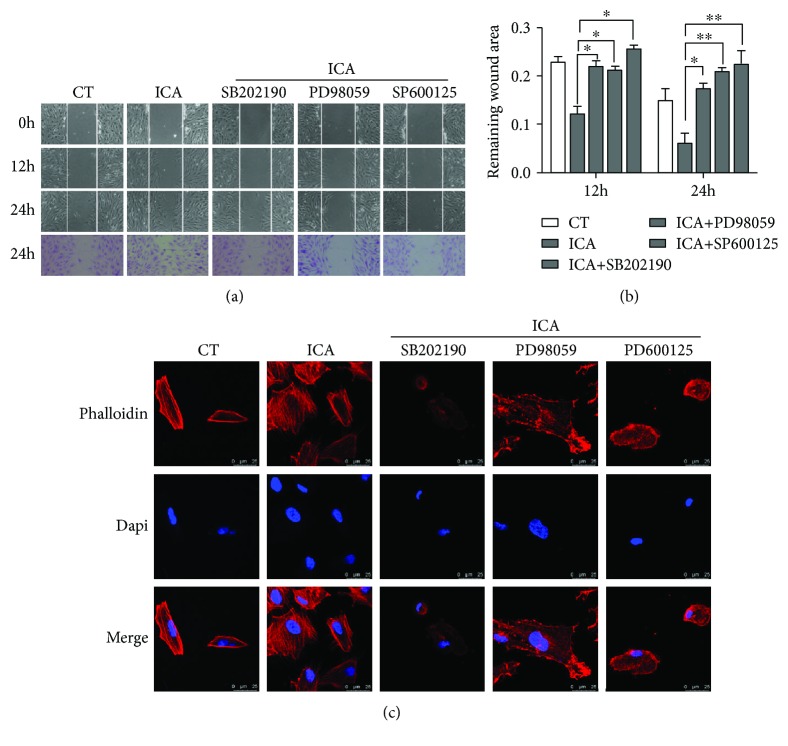
Effect of MAPK inhibitors on the wound-healing assay and rhodamine-phalloidin staining to determine the role of the MAPK signaling pathway in ICA-induced migration. (a) Activation of the MAPK signaling pathway was required for ICA-induced BMSC migration in the scratch wound-healing assay. BMSCs were treated with ICA (1 *μ*M) in the presence of three inhibitors. The wounds were evaluated at 12 and 24 h after scratching. (b) Statistical data analysis of the remaining wound area. (c) ICA induces actin stress fiber formation by upregulating the MAPK signaling pathway. BMSCs were pretreated with the three inhibitors for 1 h. Then, rhodamine-phalloidin staining was performed after treatment with ICA.

To further determine the role of the MAPK signaling pathway in the migration of BMSCs induced by ICA, migration was analysed by a scratch wound-healing assay and rhodamine-phalloidin staining in the presence of the P38-specific inhibitor SB202190, the ERK-specific inhibitor PD98059, or the JNK-specific inhibitor SP600125. As shown in Figure 5(a), in the scratch wound-healing assay, the inhibitor groups exhibited significantly reduced ICA-induced migration of BMSCs. Similar results were also obtained for rhodamine-phalloidin staining. After treatment with ICA in the presence of the three inhibitors, BMSCs had less actin stress fiber formation (Figure 5(b)).

### 3.7. ICA Improves the Homing Rates of BrdU-Labeled BMSCs

**Figure 6:**
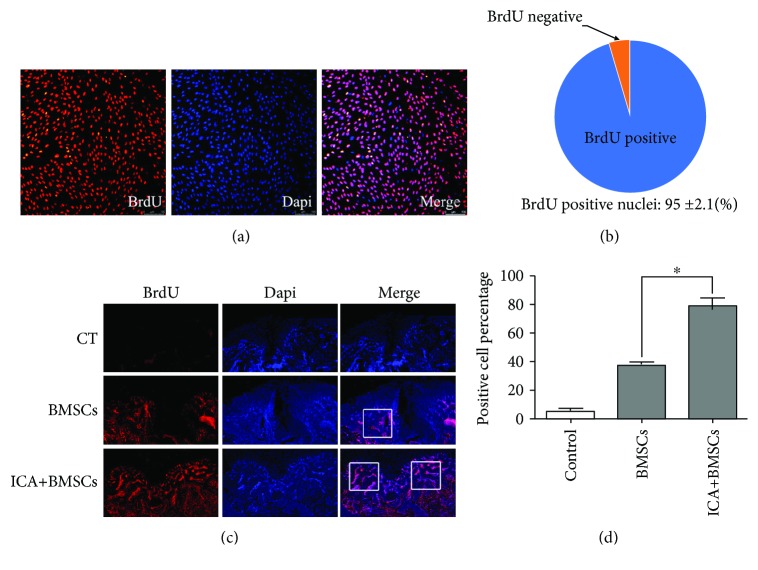
The migration of BrdU-labeled BMSCs in vivo. (a) To observe the migration of BMSCs in vivo, BMSCs were labeled with BrdU. All cells stained with DAPI were shown in blue, while BrdU-positive cells were labeled with red fluorescence. (b) The labeling efficiency of BrdU was 95 ± 2.1%. (c) BrdU^+^ cells were detected by an immunofluorescence assay at 4 weeks after surgery. The number of BMSCs treated with ICA in the repairing tissue is much greater than that in the group injected with control BMSCs. (d) Statistical data analysis of the positive cell percentage in Figure 6(c). ^∗^*P* < 0.05 compared with the BMSC control.

To ensure that most of the BMSCs were labeled by BrdU, an immunofluorescence assay was performed. BMSCs were cultured with 10 *μ*M BrdU for 72 h. Positive cells were labeled with red fluorescence in the nucleus, and the percentage of BrdU-positive cells was 95 ± 2.1% (Figures 6(a) and 6(b)). The BrdU^+^ cells in the tissues undergoing repair were detected by an immunofluorescence assay at 4 weeks after surgery. As shown in Figure 6(c), there were more BrdU-positive cells in the group that received ICA-treated BMSCs than in the group that received control BMSCs, suggesting that the combination of ICA and BMSCs led to improved migration. Moreover, the distribution of BrdU^+^ cells in the group that received ICA-treated BMSCs was more extensive and uniform than that in the group that received control BMSCs.

## 4. Discussion

With the development of cell-based therapies, BMSCs have attracted the attention of researchers for the treatment of OA. As the ideal seed cells for tissue engineering, BMSCs play important roles in the rehabilitation and regeneration of tissue. BMSCs not only have extensive proliferative ability but also retain multilineage mesenchymal differentiation potential [3, 20]. However, the restorative effect of BMSCs is determined by their homing rate, and these cells generally showed limited engraftment upon in vivo implantation due to the hostile microenvironment within the injured tissue [21, 22]. Therefore, increasing the homing rate of BMSCs should improve their therapeutic effects. In the present study, we hypothesized that ICA might promote cell migration. To determine the optimal concentration of ICA for the induction of BMSC migration, concentrations from 0.01 *μ*M to 100 *μ*M were tested in the CCK-8 assay. We found that ICA had no positive effect on BMSC proliferation, which conflicts with other reports [23], most likely due to differences in the species from which the tested cells were obtained. The wound-healing assay and Transwell migration assay showed that 1 *μ*M ICA induced more BMSC migration than other concentrations of ICA. We further showed that ICA promoted the migration of BMSCs probably by stimulating actin stress fiber formation.

Although the underlying mechanism of BMSC migration has not yet been clarified, multiple cell signaling pathways have been implicated in the regulation of BMSC migration. The MAPK signaling pathway plays a key role in the process of BMSC migration [24]. The MAPK protein family includes ERK, p38 kinase, and JNK. Extensive evidence has shown that changes in osmotic stress, heat shock, and proinflammatory cytokines can activate the MAPK signaling pathway [10]. When activated, MAPK signaling can enhance myosin light-chain kinase (MLCK) activity, which leads to increased MLC phosphorylation. The phosphorylation of MLC is associated with actin stress fiber formation in the cell body [12]. Our in vitro results suggested that ICA enhanced ERK, p38 kinase, and JNK phosphorylations, and inhibition of them decreased BMSC migration and actin stress fiber formation. These data further verified the complex pleiotropic mechanisms by which BMSC migration is regulated.

To date, many strategies have been developed to improve the homing of BMSCs to the injured site [25–27]. First, BMSCs were genetically engineered to change the genotype of its progeny to improve their homing ability [28]. Second, cytokines were used to induce homing receptor expression in BMSCs to promote their migration [29]. Third, a magnetic system was designed to guide the superparamagnetic iron oxide nanoparticle- (SPION-) labeled cells precisely to the lesion location [30]. However, the safety of the first two methods remains doubtful due to the varying effects [31, 32], and the weakness of the third method is that SPION can cause oxidative damage in tissues. Compared with other strategies, ICA treatment, which by itself was reported to accelerate the formation of cartilage matrix and chondroid tissue, could exert positive effects on BMSCs [19, 23, 33]. In the present study, we successfully labeled the BMSCs with BrdU. The immunofluorescence assay of repairing tissues with the participation of the BrdU-labeled BMSCs showed that ICA could increase the recruitment of BMSCs into the cartilage defect region. Moreover, the distribution of BrdU^+^ cells in the group that received ICA-treated BMSCs was more extensive and uniform than that in the group that received control BMSCs. This finding demonstrates that ICA-treated BMSCs may become a very promising strategy for the repair of cartilage defects and will increase the possibilities for treatment of other diseases requiring high homing rates.

Although the repairing effect of ICA combined with BMSCs was not detected, the gross appearance showed better results. In future studies, the long-term effects of transplanted ICA-treated BMSCs should be investigated in more detail.

## 5. Conclusion

In summary, the data reported herein show that ICA promotes BMSC migration in vivo and in vitro. In addition, the mechanism of ICA-induced BMSC migration involves the promotion of actin stress fiber formation via the MAPK signaling pathway. Hence, combined therapy of BMSCs with ICA may confer better results in the treatment of cartilage defects and may be a challenging direction for further study.

## Data Availability

The data used to support the findings of this study are available from the corresponding author upon request.
